# Six Rotation Years Drive Dynamic Shifts in Agronomic Traits, Photosynthesis, and Tuber Metabolomics of *Dioscorea opposita* Thunb

**DOI:** 10.3390/metabo16070492

**Published:** 2026-07-13

**Authors:** Yange Yu, Dandan Dai, Guixiao La, Xiangyang Li, Xiaoyang Guo, Yi Wen, Tiegang Yang

**Affiliations:** 1 Institute of Chinese Herbal Medicines, Henan Academy of Agricultural Sciences, Zhengzhou 450002, China; 2 Henan Provincial Engineering Technology Research Center of Chinese Medicinal Materials for Genuineness, Zhengzhou 450002, China; 3 Institute of Tropical Crops Genetic Resources, Chinese Academy of Tropical Agricultural Sciences, Haikou 571101, China

**Keywords:** *Dioscorea opposita* Thunb., Chinese yam, rotation years, metabolomics, flavonoids, photosynthetic activity

## Abstract

**Background/Objectives:** *Dioscorea opposita* Thunb. is widely cultivated as a nutritious crop; however, the dynamic effects of different rotation years on its agronomic traits, photosynthetic performance, and tuber metabolome remains poorly understood. This study aimed to systematically investigate the effects of varying rotation years on these parameters. **Methods**: Six field treatments were established: Y0 (0-year rotation, continuous cropping), Y1 (1-year), Y2 (2-year), Y3 (3-year), Y4 (4-year), and CK (fallow land with ≥8 years of rotation). Tuber metabolomic profiling was performed using UPLC-MS/MS. **Results**: Rotation years <4 showed no differences in tuber length or yield, with tuber length ranging from 35.42 to 47.18 cm and yield from 103.31 to 133.77 g. Shorter rotations (<4 years) exhibited significantly reduced chlorophyll content, as well as diminished photosynthetic parameters (*Pn*, *Gs*, and *Tr*). Metabolomic analysis identified 93 metabolites, of which 30 were differential, with 11 showing enrichment in Y4. Notably, procyanidin B1, kaempferol, dihydroquercetin, pyridoxine, and L-cystine exhibited higher levels in Y0. Sinapic acid, tyramine, glutamine, dibutyl phthalate, and fructose reached peak levels in fallow land (CK). All pairwise comparisons except Y4 vs. CK were enriched in the flavonoid and flavone biosynthesis pathway, with flavonoids representing the predominant class of differential metabolites. Cluster analysis showed that metabolites in clusters 1 and 7 peaked at Y4, while those in clusters 3, 4, 5, and 6 reached minimum levels at Y4. **Conclusions**: Rotation years of less than 4 years led to restricted growth, reduced yield, impaired chlorophyll synthesis, and altered metabolic profiles. A four-year rotation is identified as a critical turning point in metabolite accumulation, providing valuable guidance for sustainable *D. opposita* cultivation.

## 1. Introduction

The concept of medicine–food homology, which integrates dietary foods and traditional Chinese medicine principles, has attracted increasing attention in recent years [1]. *D. opposita* (*Dioscorea opposita* Thunb.), commonly known as “shanyao” or Chinese yam, was one of the candidates for medicine-food homology published by China in 2002 [2]. *D. opposita* was first documented in Shennong’s Herbal Classic (the earliest extant comprehensive pharmacological treatise in China, systematically compiling pre-Qin and Han dynasty medical knowledge) [3], and has a long history of cultivation and medicinal use [4]. It is widely cultivated and distributed across China, mainly in the northeastern, central, and southeastern regions [5]. According to Ben Cao Gang Mu, *D. opposita* tuber exhibits remarkable therapeutic efficacy in spleen-stomach tonification, immune enhancement and anti-aging applications [6]. The tuber contains a variety of nutritional and functional metabolites, such as amino acids, starch, polysaccharides, flavonoids, polyphenols, dopamine, saponins, batatasins, and other pharmacologically active compounds [7,8]. These metabolites possess antioxidant, anti-tumor, anti-inflammatory, cardiovascular-protective, and anti-aging activities [2,9], which collectively contribute to the medicinal effects of *D. opposita* [10]. Consequently, the chemical composition of *D. opposita* critically determines its quality and functional attributes.

Extensive research has been conducted to evaluate the effects of anthropogenic factors on the growth and quality of *D. opposita* during cultivation. Key determinants include cultivation patterns [11], sowing densities [12], irrigation regimes [13], fertilizer application strategies [14], plant growth regulators [15], and harvest timing [9]. Beyond these agronomic interventions, soil plays a critical role in regulating plant growth, development and secondary metabolism [16]. Soil composition affects agronomic traits and quality parameters [17], while distinct soil types mediate differential effects on plant growth and secondary metabolite accumulation [18]. Building on these, recent studies have focused on morphology variation and the profiles of primary and secondary metabolites (e.g., total polysaccharides, amino acids, proteins, flavonoids, polyphenols and saponins) in *D. opposita* grown in contrasting soil types, including sandy soil and loessial soil [4,17].

Notably, soils with different crop rotation years have been shown to influence plant growth and quality [19]. Prolonged rotations enhance soil organic carbon storage and sustain soil biological activity, thereby promoting plant development [20]. Crop rotation is also an effective strategy for overcoming continuous cropping obstacles. Such obstacles are commonly encountered in *D. opposita* cultivation due to large-scale planting and limited arable land [21]. In contrast to continuous cropping, rotation effectively suppresses plant diseases, improves nutrient availability, and ultimately enhances crop growth and yield [22]. For example, rotation with rice has been demonstrated to significantly alleviate continuous cropping obstacles in “multi-functional lily” by restructuring rhizosphere microbial communities and promoting secondary metabolite accumulation [23]. In ginseng cultivation, rotating with *Amorpha*, alfalfa and *Asarum* species has shortened the rotation interval from 20~30 years to 6~10 years [24]. Similarly, *Radix pseudostellariae* faces serious continuous cropping obstacles, with yield and quality recovery typically requiring a fallow period of 3–4 year before replanting in the same field. Among various strategies, rice-paddy-upland rotation is regarded as the most effective intensive ecological approach for mitigating continuous cropping obstacles in *Radix pseudostellariae* [25].

However, to date, comprehensive studies elucidating the effects of different rotation years on the growth performance, photosynthetic activity, and quality parameters of *D. opposita* remain limited. A particularly critical research gap exists concerning the physiological mechanisms governing morphological adaptation and metabolite accumulation in *D. opposita* under varying rotation years. Under conventional practice, a five-year fallow period is generally required before *D. opposita* can be replanted on the same land [26]. To systematically investigate the effects of rotation years on *D. opposita* cultivation, six field treatments were established: Y0 (0-year rotation, continuous cropping), Y1 (1-year rotation), Y2 (2-year rotation), Y3 (3-year rotation), Y4 (4-year rotation), and CK (fallow land with ≥8 years rotation, serving as a control for *D. opposita* growth). This study had two primary objectives: (1) to assess the effects of different rotation years on the agronomic traits, photosynthetic activity and tuber metabolite profiles of *D. opposita*; and (2) to elucidate the physiological mechanisms underlying photosynthetic adaptation and metabolite regulation in response to different rotation regimes, with a particular emphasis on characterizing the dynamic changes in key bioactive compounds. To achieve these objectives, comprehensive metabolite profiling was performed across rotation treatments, enabling systematic identification of rotation-specific metabolite signatures and their correlation with agronomic performance. The findings will provide a robust scientific foundation for a systematic and comprehensive analysis of the impact of different rotation years on *D. opposita*. This analysis will offer valuable insights into the development of sustainable cultivation practices in regions where *D. opposita* is produced.

## 2. Materials and Methods

### 2.1. Experimental Field Design

A field experiment was conducted at the *Dioscorea opposita* resource base of the Collaborative County-Institute in Mapo Village, Qinyang, Jiaozuo City, Henan Province, China. Qinyang, historically designated as *Henei* County and serving as the administrative center of ancient *Huaiqingfu*, is renowned as the traditional production area for *Dioscorea opposita*. The experimental site is situated at the intersection of the Yellow River alluvial plain and the southern foothills of the Taihang Mountains. According to regional soil survey data [27], the soil composition in this area primarily comprises alluvial deposits derived from the Yellow River and its tributary, the Qin River. The field experiment began in April 2019 and ended in November 2024, encompassing six treatments and six rotation years: Y0 (0-year rotation, continuous cropping), Y1 (1-year rotation), Y2 (2-year rotation), Y3 (3-year rotation), Y4 (4-year rotation), and CK (uncultivated *D. opposita* land with rotation years ≥ 8). In the Y0 treatment, yams were planted in 2023 following continuous cultivation. The Y1 treatment involved a one-year rotation cycle of wheat and corn after yam cultivation in 2022. Similarly, the Y2 treatment implemented a two-year wheat–corn rotation following the 2021 yam harvest. The Y3 treatment utilized a three-year rotation of wheat and corn after the 2020 yam season. The Y4 treatment employed a four-year wheat–corn rotation after the 2019 yam harvest. The CK treatment served as a control, with initial sowing of wheat and corn, but never cultivated for yam. Six replications were performed for each treatment. The experimental design and treatment configurations were visually presented in Figure S1.

The cultivar *Dioscorea opposita* Thumb. cv. Tiegun was employed for cultivation. Healthy and morphologically uniform rhizomes of this variety were selected and transplanted into the experimental plots in early April 2024 for all rotation treatments. Each plot size was 10 m × 3.5 m with 6 rows, a row-to-row distance of 70 cm, and a planting distance of 20 cm. All plots were treated with identical management practices. Specifically, no fertilizers, pesticides, or weeding were applied during the yam cultivation period. According to the above planting pattern, *D. opposita* growth cultivated in the fields adjacent to different rotation years was also observed.

### 2.2. Plant Agronomic Trait Assessment and Disease Incidence Observations

Following the method of Silva et al. [28], harvest was conducted when aboveground vines had fully senesced (yellowing and drying). At this uniform stage, 20 plants were randomly selected from each plot on 23 November 2024. Tuber length, diameter, and fresh weight were recorded. Length and diameter were measured using a millimeter scale and vernier caliper. The tuber fresh weight per plant was recorded as *D. opposita* yield. Fresh tubers from each replicate were washed with distilled water, cut up and mixed together as one biological replicate, then oven-dried at 55 °C until constant weight.

A general foliar disease index was used to assess disease severity in *D. opposita* at the maturity stage. Disease symptoms were defined as visible necrotic lesions with clear margins, typically accompanied by chlorosis or defoliation. The disease index (DI) was classified into five grades: Grade 0 (healthy): no lesions on any leaves; Grade I (mild): 1–5% of leaf area with lesions; Grade II (moderate): multiple lesions on lower leaves and a few on upper leaves, with 5–20% defoliation; Grade III (severe): extensive lesions with 20–70% defoliation; Grade IV (critical): >70% defoliation or plant death.

To ensure accuracy, symptoms from nutrient deficiency (chlorosis without lesions), insect damage (holes or insect presence), and physiological disorders (lesion-free wilting or curling) were excluded. Only clear necrotic lesions were scored as disease. During the survey, 20 plants were examined per plot. The disease index were calculated as follows [29]:

$$Disease\;index=\frac{\sum (Disease\;level\times Number\;of\;plants\;at\;that\;level)}{Total\;number\;of\;plants\;surveyed\times Highest\;disease\;level}\times 100$$

### 2.3. Photosynthesis Determination

To monitor changes in the chlorophyll content and photosynthetic parameters of *D. opposita* leaves under six different rotation years, leaf samples were collected at multiple time points after planting: 60, 90, 120, 150 and 180 days after planting (DAP). At each time point, six plants were randomly selected from each replicate, and three mature, functional leaves per plant were measured at the same position. Chlorophyll content was measured using a chlorophyll meter (SPAD-502, Konica Minolta, Tokyo, Japan). Photosynthetic parameters of net photosynthetic rate (*Pn*, µmol CO_2_ m^−2^ s^−1^), stomatal conductance (*Gs*, mol H_2_O), transpiration rate (*Tr*, mmol H_2_O) were measured using a portable photosynthesis system (CIRAS-3, Hansatech Ltd., Norwich, UK). Measurements were taken between 9:00 and 11:00 on a sunny day, under an ambient CO_2_ concentration of 380 µmol mol^−1^, with a leaf temperature of 25 ± 1 °C, a photosynthetic photon flux density of 1000 µmol m^−2^ s^−1^ and a relative air humidity of 70–80% in the leaf chamber.

### 2.4. Sample Preparation

The preparation of *D. opposita* tuber samples was performed according to previously published methods [30] with minor modifications to optimize for metabolomic analysis. The oven-dried (55 °C) tubers were pulverized using a high-speed grinder and passed through a No. 60 mesh sieve (particle size < 0.25 mm) to ensure homogeneity.

For each biological replicate, approximately 0.30 g (accurately weighed to 0.001 g) of the powdered sample was transferred into a 50 mL centrifuge tube. Extraction was carried out by ultrasonication (40 kHz, 300 W) in an ice-water bath (maintained at 4 °C) 60 min using 15 mL of 75% (*v/v*) aqueous methanol. Following extraction, the mixture was centrifuged at 12,000 rpm at 4 °C for 10 min. An aliquot of 1.5 mL of the supernatant was filtered through a 0.22 µm membrane into an amber vial for UPLC-MS/MS analysis.

Samples from different rotation treatments (Y0, Y1, Y2, Y3, Y4, and CK) were extracted in three independent biological replicates (*n* = 3) to assess methodological reproducibility. A quality control (QC) sample was prepared by pooling equal aliquot of all sample extracts and was injected after every 10 samples to monitor instrument stability.

### 2.5. UPLC-MS/MS Analysis

Chromatographic separation was performed on a Welch Ultimate XB-C18 column (2.1 mm × 100 mm, 1.8 µm). The gradient elution system consisted of (A) 0.1% formic acid in water and (B) 0.1% formic acid in acetonitrile as the mobile phase. The elution program was set as follows: 0–1.5 min, 5–10% B; 1.5–5 min, 10–65% B; 5–6 min, 65–100% B; 6–8 min, 100% B; 8–8.1 min, 100–5% B; 8.1–11 min, 5% B. The flow rate was 0.4 mL/min, the injection volume was 5 μL, and the column temperature was maintained at 40 °C.

Mass spectrometry (MS) analysis was performed on a Waters Xevo G2 QTOF/MS (Waters Corp., Milford, CO, USA). The samples were detected in the positive and negative ion modes using an electrospray ionization (ESI) source. The mass spectrometry scanning range was set to 50–1200 *m*/*z*. The optimized conditions were as follows: ion spray voltage: 5.0 kV (ESI+) and 4 kV (ESI−); cone voltage: 60 V; cone gas flow rate: 50 L/h; source temperature: 110 °C.

### 2.6. Statistical Analysis

Data analysis was performed using R software (version 4.3.1, https://www.r-project.org/, accessed on 12 December 2025). Principal component analysis (PCA) was conducted on the Metware Cloud, a free online data analysis platform (https://cloud.metware.cn, accessed on 12 December 2025) [3]. Differential metabolites were identified based on the following criteria: a variable importance in the projection (VIP) value of greater than 1 (VIP *>* 1) and an adjusted *p*-value of less than 0.05 (*p* < 0.05). One-way analysis of variance (ANOVA) followed by Tukey’s post hoc test was performed using GraphPad Prism (version 8.0.1) to determine statistically significant differences among groups. Metabolic pathway annotation of the differential metabolites was carried out using the Kyoto Encyclopedia of Genes and Genomes (KEGG) database. Furthermore, the R software package was utilized to generate additional graphical representations.

## 3. Results

### 3.1. Variations in Morphology, Yield, and Disease Incidence of D. opposita Across Different Rotation Years

**Figure 1:**
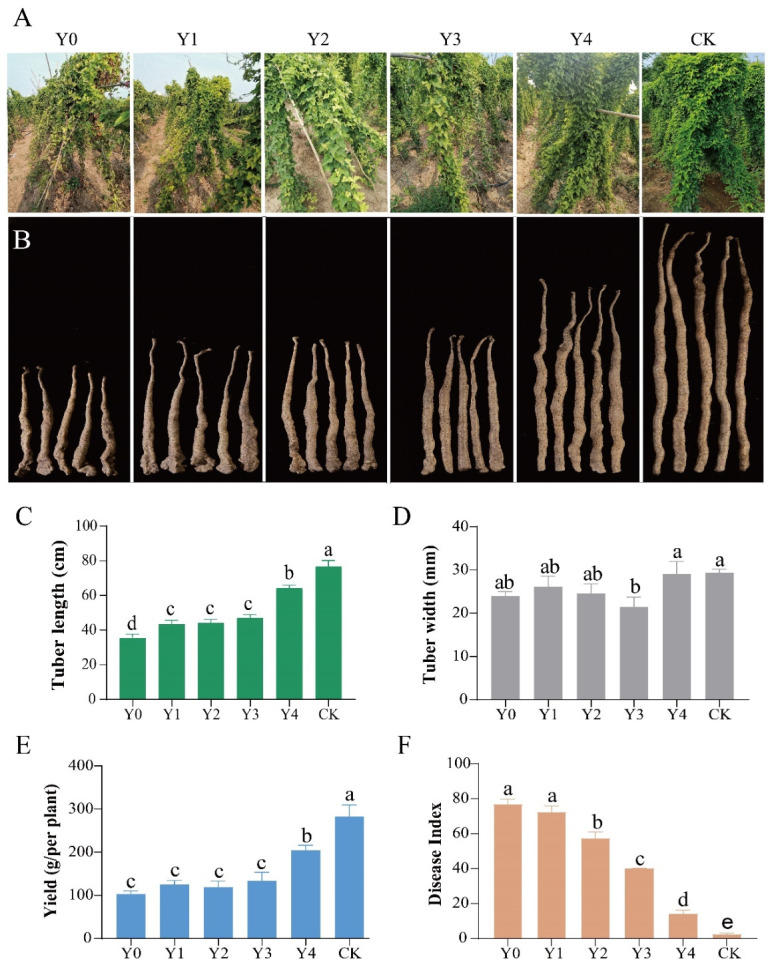
Influences of plants growth across six rotation years (Y0, Y1, Y2, Y3, Y4 and CK) in *D. opposita*. (**A**) Morphology of the above-ground part; (**B**) morphology of the tuber; (**C**) tuber length (means ± SD, *n* = 20); (**D**) tuber width (means ± SD, *n* = 20); (**E**) yield; (**F**) disease index. Different letters indicate significant differences at *p* < 0.05.

The morphological phenotypes of *D. opposita* cultivated in soil with different rotation years were evaluated. As shown in Figure 1A,B, samples designated Y0, Y1, Y2, Y3, Y4, and CK displayed distinct morphological variations corresponding to increasing rotation years. Specifically, the above-ground and tuberous portions of *D. opposita* plants cultivated under the CK displayed healthy and robust foliage, with the Y4 treatment exhibiting comparable characteristics. In contrast, as the rotation years decreased, plants in the Y0, Y1, Y2, and Y3 fields exhibited chlorotic leaves accompanied by lesions, alongside stunted, forked, and malformed tubers. Moreover, plants grown under Y3 showed less robust in comparison to those grown under Y4.

Concurrently, substantial variations in agronomic traits (i.e., tuber length, tuber diameter, and yield) were observed across different crop rotation years (Figure 1C–E). Tuber length and yield differed significantly among rotation treatments (*p* < 0.01). The lowest value was observed in Y0, while CK was the highest. Y0 tubers showed the shortest average length (35.42 cm), followed by Y1 (43.47 cm), Y2 (44.25 cm), and Y3 (47.18 cm). No significant differences were observed among these four treatments. Conversely, Y4 tubers exhibited a marked increase in length (66.22 cm), with the longest tubers observed in CK (76.81 cm; see Figure 1C). Tuber width remained relatively stable across all rotation years, ranging from 21.46 to 29.31 mm. However, the tuber width of Y3 (21.46 mm) was significantly lower than that of other rotation years (see Figure 1D). Similarly to tuber length, tuber yield increased progressively with prolonged rotation years in the order: Y0 < Y2 < Y1 < Y3 < Y4 < CK. The yields of Y0 (103.31 g), Y1 (125.26 g), Y2 (118.96 g), and Y3 (133.77 g) did not differ significantly. In contrast, the yields of Y4 (214.73 g) and CK (282.27 g) were significantly higher (Figure 1E).

Disease incidence varied markedly among the six crop rotation treatment (Figure 1F). As the years of rotation increased, the disease severity showed a progressive decline. The incidence rate of *D. opposita* was significantly higher in Y0 and Y1 than in other treatments, with no statistically significant difference between these two groups. The disease index decreased from 77.00 in Y0 to 2.62 in CK. The disease index of Y4 (14.05) was significantly higher than that of Y3 (40.14) but remained lower than the CK value. Specifically, Y3 and Y4 plants exhibited disease severity levels classified as grade III and grade II, respectively. Y2 showed a considerably higher disease index (57.52) than Y3, although both fell into grade III severity.

In summary, tuber length and yield of *D. opposita* increased progressively with extended rotation years, with Y0 exhibiting the lowest values. Y0 and Y1 showed the highest disease indices (indicating the most severe disease levels), whereas Y4 and CK demonstrated the lowest severity. Specifically, the above-ground growth, tuber length, and tuber yield in Y4 exhibited statistically significant increases (*p* < 0.05) compared to Y0, Y1, Y2, and Y3 treatments. However, these parameters remained lower than those observed in CK. Consequently, Y4 delineates a critical temporal threshold in this cropping system, beyond which further rotation benefits plateaued while disease suppression continues to be effective.

### 3.2. Photosynthetic Characteristics of D. opposita Across Different Rotation Years

**Figure 2:**
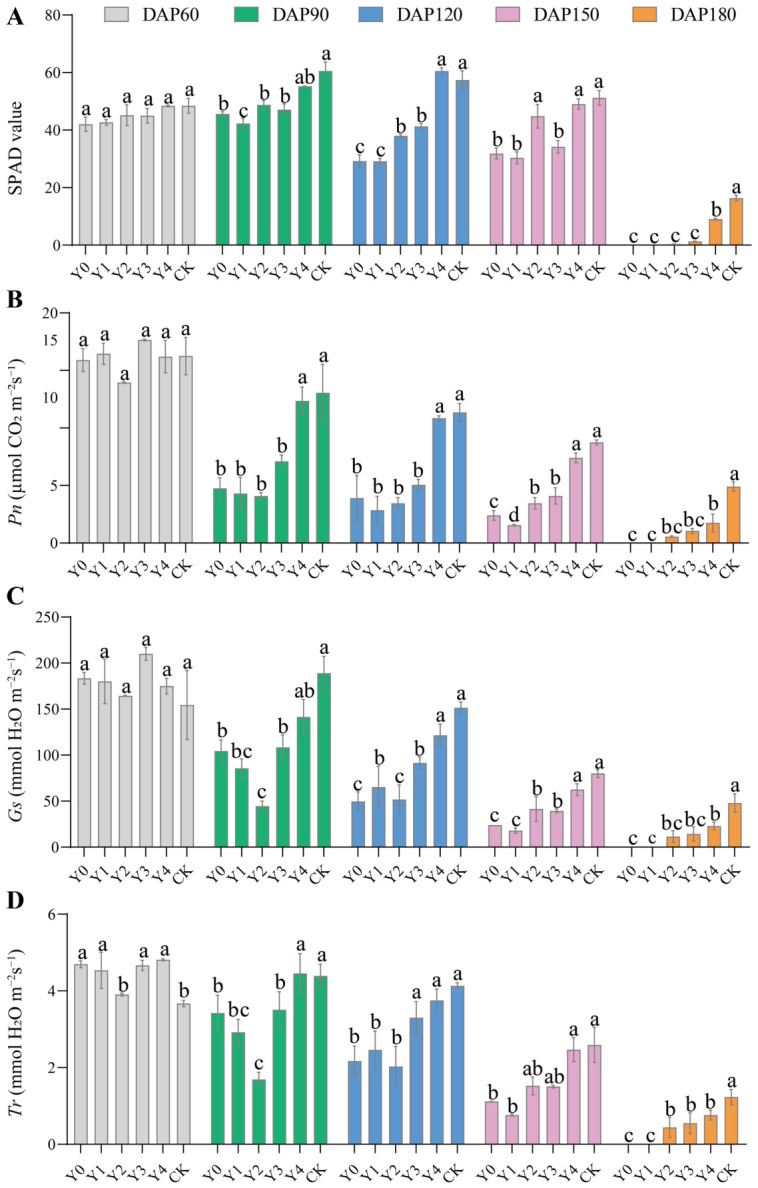
Dynamics of chlorophyll content (SPAD value) in *D. opposita* leaves across six different years of rotation during five growth stages (**A**), and photosynthetic characteristics *Pn* (**B**), *Gs* (**C**), and *Ci* (**D**). Note: DAP 60 (60 days after field planting); DAP 90 (90 days after field planting); DAP 120 (120 days after field planting); DAP 150 (150 days after field planting); DAP 180 (180 days after field planting). Different letters indicate significant differences at *p* < 0.05.

The present study demonstrated that rotation years significantly affected the daily fluctuations in chlorophyll content (SPAD values), net photosynthetic rate (*Pn*), stomatal conductance (*Gs*), and transpiration rate (*Tr*) of *D. opposita* across five growth stages (60–180 days after planting, DAP). Figure 2A shows that the chlorophyll content in yam leaves varied among the six rotation treatments over time. At 60 days after field planting (DAP 60), chlorophyll content did not exhibit significant differences among the six rotation years. From DAP 90 to DAP 150, Y4 and CK consistently exhibited the highest chlorophyll content, with no significant differences between them. At DAP 90, Y1 showed the lowest chlorophyll content (42.30) among all treatments. Between DAP 120 and DAP 150, Y0, and Y1 maintained the lowest levels (29.14–31.86). At DAP 150, Y3 showed an intermediate value (34.15), while CK reached the highest (51.20), followed by Y4 (49.05). At DAP 180, no chlorophyll content was detectable in Y0, Y1, and Y2, as the leaves had fully shed. Y3 exhibited minimal chlorophyll content (1.30) with severe leaf loss, while Y4 and CK showed yellowing leaves with values of 9.00 and 16.40, respectively. Collectively, after DAP 90, plants with shorter rotation years (Y0, Y1, Y2, and Y3) displayed progressively declining chlorophyll content. By DAP180, plants cultivated for fewer than four years (Y0, Y1, Y2, and Y3) had undergone complete aboveground leaf desiccation and abscission.

Similarly, at DAP 60, *Pn* exhibited a no significant variation across rotation years (Figure 2B). The *Pn* values ranged from 13.90 to 16.50 µmol m^−2^ s^−1^. Between DAP 90 and 120, *Pn* remained consistently low in Y0, Y1, Y2 and Y3 (2.85–7.10 µmol m^−2^ s^−1^). Y4 and CK maintained higher levels (10.85–14.8 µmol m^−2^s^−1^). At DAP 150, *Pn* values across the six treatments followed the order: Y2 < Y0 < Y1 < Y3 < Y4 < CK. By DAP 180, *Pn* was undetectable in Y0 and Y1, while CK exhibited the highest value (4.90 µmol m^−2^s^−1^). No significant differences were observed among Y2, Y3, and Y4, with values ranging from 0.55 to 1.75 µmol m^−2^ s^−1^.

Furthermore, *Gs* and *Tr* showed similar trends to *Pn* (Figure 2C,D). At DAP 60, *Gs* showed no significant variation across the six rotation years, while *Tr* values were significantly lower in Y2 and CK. At DAP 90, the lowest *Gs* and *Tr* values were observed in Y2, followed by Y1 and then Y0. At DAP 120, *Gs* in Y3 differed from that in Y4 and CK, while *Tr* showed no significant correlation between Y4 and CK. Both *Gs* and *Tr* in Y3 were higher than in Y0, Y1, and Y2 but lower than in Y4 and CK. From DAP 150 to DAP 180, both parameters showed a clear increasing trend: Y1 < Y0 < Y2 < Y3 < Y4 < CK. As with *Pn*, *Gs,* and *Tr* were undetectable in Y0 and Y1 at DAP 180 due to leaf wilting, yellowing, and abscission.

### 3.3. Metabolomic Profiling Analysis of D. opposita Across Different Rotation Years

To assess the metabolic changes in *D. opposita* tubers cultivated under six rotation years (Y0, Y1, Y2, Y3, Y4, and CK), untargeted metabolomic profiling was conducted using UPLC-MS/MS. In the present study, 93 metabolites were identified across all samples, comprising 18 amino acid and derivatives, 14 saccharides, 13 phenolic acids, 10 organic acids, 9 alkaloids, 9 flavonoids, 5 nucleotides and derivatives, 5 vitamins, 4 steroids, and 6 other compounds (Table 1 and Figure S2A). The top six categories were amino acid and derivatives (19.35%), saccharides (15.05%), phenolic acids (13.98%), organic acids (10.75%), alkaloids (9.68%), and flavonoids (9.68%). These metabolites collectively constitute the primary nutritional attributes of *D. opposita*.

Principal component analysis (PCA) was conducted to visualize clustering patterns among the treatment groups. In the PCA score plot, each data point corresponds to an individual sample, with spatial distribution reflecting similarities or differences in overall metabolite profiles [3]. The PCA demonstrated the separation of Y0, Y1, Y2, Y3, Y4, and CK and the clustering of the replicates (Figure S2B). The first two principal components (PC1 and PC2) accounted for 67.14% of the total variance, with PC1 and PC2 explaining 41.5% and 25.64%, respectively. In the score plot, six biological replicates from each group were clustered, indicating the repeatability and reliability of the metabolomic analysis. Six groups were divided into three categories, showing rotation years had a significant impact on metabolic profiles. CK and Y4 exhibited more distinct and independent clustering trends in comparison to the other treatment groups. The third cluster comprises Y0, Y1, Y2, and Y3. Y1 and Y2 appeared together and were adjacent to Y0 and Y3. A minor alteration in metabolites was observed from Y0 to Y3, but a significant change emerged between Y3 and Y4.

#### 3.3.1. Clustering of Metabolites by K-Means Analysis

**Figure 3:**
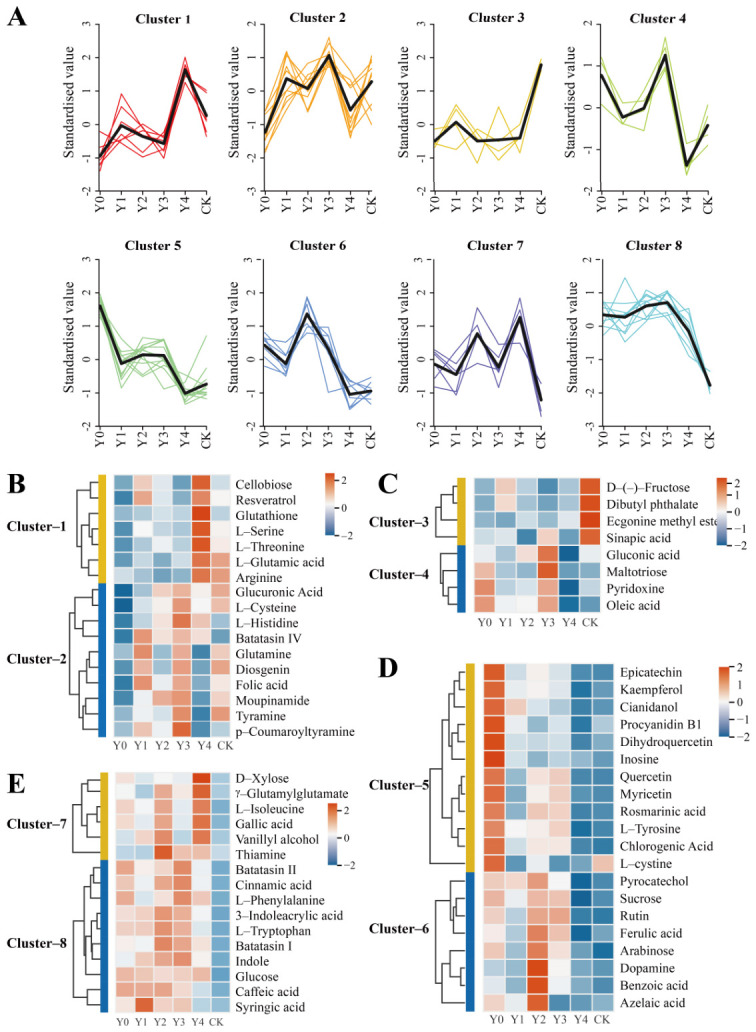
Metabolite analysis of *D. opposita* in Y0, Y1, Y2, Y3, Y4, and CK. K-means clustering analysis of metabolites (**A**). Visual analysis of heatmap clustering for Cluster 1–8 (**B**–**E**). Note: The heatmap displays metabolite distributions across different clusters, with color-coding to indicate expression levels from low (blue) to high (red).

K-means clustering was employed to delineate the accumulation patterns of metabolites across six rotation years. The investigation revealed that 93 metabolites were grouped into eight clusters (Figure 3A) based on the similarity of metabolite changes. The relative content trends for each cluster are visualized as heatmaps in Figure 3B–D. Cluster 1 consisted of seven metabolites, including five amino acid derivatives, resveratrol, and cellobiose, which exhibited the lowest levels at Y0 and reached a peak at Y4. Cluster 2 comprised 10 metabolites, with a marked increase at Y3. Alkaloid compounds such as tyramine, moupinamide, and p-coumaroyltyramine were grouped together, which were highly abundant in Y3 but were scarce in Y4. Diosgenin demonstrates a comparable tendency in its concentration fluctuation. In Cluster 3, the levels of sinapic acid, dibutyl phthalate, fructose and ecgonine methyl ester were found to be highest in the CK group. In comparison with CK, their levels remained consistently lower from Y0 to Y4. Cluster 4 comprised four metabolites, with the highest levels observed in the Y3 group and the lowest levels observed in the Y4 group.

Cluster 5, the largest flavonoid-dominated group with 12 metabolites, displayed a progressive decline in content with increasing rotation years. Levels of these metabolites reached a maximum at Y0, subsequently plateaued during Y0–Y3, and then dropped sharply between Y3 and Y4. Clusters 6 and 7 comprised 8 and 6 metabolites, respectively, both showing the lowest levels in CK. Metabolites in Cluster 6 increased notably at Y2. Cluster 7 demonstrated a fluctuating pattern and reached their maximum levels at Y4. These two clusters included phenolic acids (i.e., gallic acid, ferulic acid and pyrocatechol), saccharides (i.e., arabinose, sucrose and xylose) and dopamine. Finally, Cluster 8 contained 10 metabolites, including three phenolic acids (syringic acid, caffeic acid, cinnamic acid), two amino acids (L-phenylalanine and L-tryptophan), two steroidal compounds (batatasin I and batatasin II), glucose, and so on. The metabolites exhibited a downward trend from Y3 to CK, reaching their nadir at CK. The findings obtained from this study offer valuable insights into the metabolic shifts that occur with rotation years in yam, highlighting specific metabolites and their temporal patterns. Of particular note, Cluster 5 metabolites (predominantly flavonoids) levels underwent a gradual decline in proportion with increasing crop rotation years. Furthermore, metabolites in Cluster 1 and 7 attained maximum levels at Y4. Conversely, metabolites in clusters 3, 4, 5 and 6 attained their lowest levels at Y4. As demonstrated in the results, Y4 is a pivotal factor and a critical turning point, as revealed by metabolite analysis.

#### 3.3.2. Logarithm of the Fold Change in Metabolites Between Two Groups

**Figure 4:**
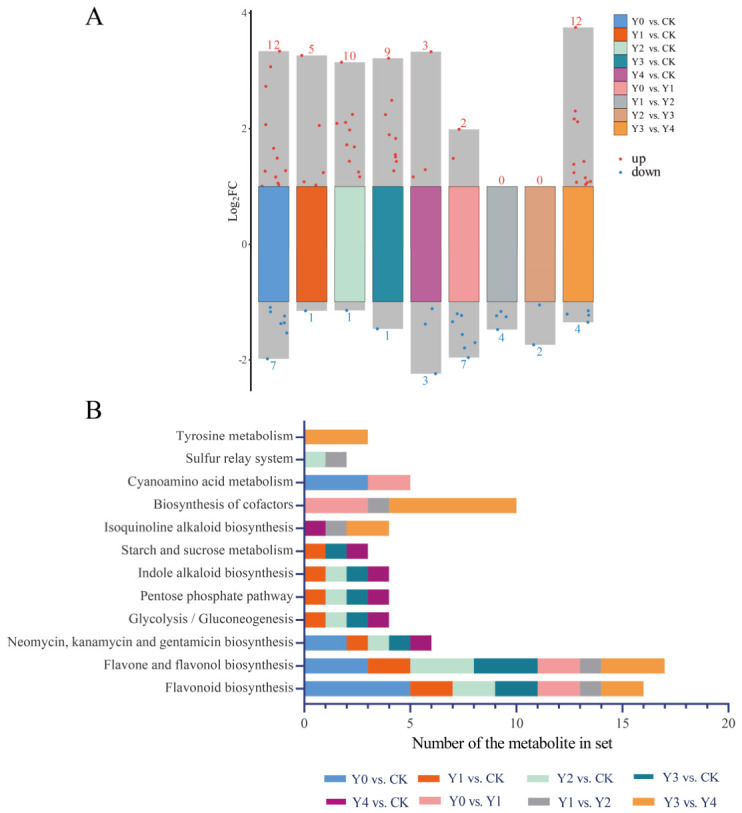
(**A**) Logarithm of the fold change in metabolite (log_2_FC) in *D. opposita* between two groups, comparing the number of up-regulated and down-regulated metabolites in each group. The criteria for significance were *p* < 0.05 and FC > 1.5. (**B**) KEGG pathway enrichment of the differential metabolites between groups. Note: Blue represents down-regulation, red represents up-regulation.

The logarithm of the fold change (log_2_FC) was calculated to quantify differential metabolites across treatment groups relative to the control (CK) in *D. opposita*. Among all groups, Y0 exhibited the highest number of differential metabolites relative to CK, with a total of 19 types, of which 12 were up-regulated and 7 down-regulated (Figure 4A). The Y1 and Y4 group showed an identical number of differential metabolites, each with six distinct metabolites. The Y2 group exhibited 11 distinct types of differential metabolites, of which 10 were up-regulated and only one down-regulated. A total of ten different types of differential metabolites were identified in the Y3 group. Except for Y4, all treatment groups (Y0, Y1, Y2, and Y3) displayed a greater proportion of up-regulated than downregulated metabolites when compared with CK. Notably, the Y0 vs. CK comparison revealed the highest number of up-regulated differential metabolites, of which 50% were flavonoids, followed by phenolic acids. A comparison of the metabolite profiles of Y4 and CK (fallow land) revealed that Y4 exhibited the least significant differences, while Y0 exhibited the most substantial deviations from the control.

Further analysis was conducted on differential metabolites between adjacent comparison groups. The number of differential metabolites identified in the pairwise comparisons Y0 vs. Y1, Y1 vs. Y2, Y2 vs. Y3, Y3 vs. Y4, and Y4 vs. CK was 9, 4, 2, 16 and 6, respectively. The number of differentially metabolites between the Y3 group and the Y4 group was the largest. It is evident that among the six different intervals treatments, Y4 represents a critical juncture and functions as a pivotal turning point. Detailed information on differential metabolites for all comparison groups was provided in Supplementary Table S1. Among the 86 differential metabolites (including duplicates) of all the comparison groups, 38 (46%) were flavonoids and phenolic acids. For instance, rutin and quercetin were both upregulated in CK in comparison to Y0, Y2 and Y3. Concurrently, cinnamic acid, a phenolic acid metabolite, showed increased expression in Y0 compared with CK, as well as in Y2 vs. CK and Y3 vs. CK.

#### 3.3.3. KEGG Pathway Enrichment of Differential Metabolites Across Rotation Treatments

To further elucidate the impact of different crop rotation years on metabolite accumulation, pathway enrichment analysis of differential metabolites across pairwise comparison groups was performed using the KEGG database (Figure 4B). The most significantly enriched metabolic pathways were flavonoid biosynthesis, flavone and flavonol biosynthesis and biosynthesis of cofactors. All of the difference combinations except Y4 vs. CK were enriched in both flavonoid biosynthesis and flavone and flavonol biosynthesis. Among these, the Y0 vs. CK comparison exhibited the highest number of differentially accumulated metabolites involved in the flavonoid biosynthesis pathway, totaling five compounds. Meanwhile, the Y3 vs. Y4 comparison displayed the greatest enrichment in the biosynthesis of cofactors, and the number of differential metabolites was six. In addition, tyrosine metabolism was individually enriched in the Y3 vs. Y4 group. These enriched metabolic pathways are closely associated with the quality variation in *D. opposita* under different crop rotation regimes.

#### 3.3.4. Heatmap of 30 Significant Metabolites

**Figure 5:**
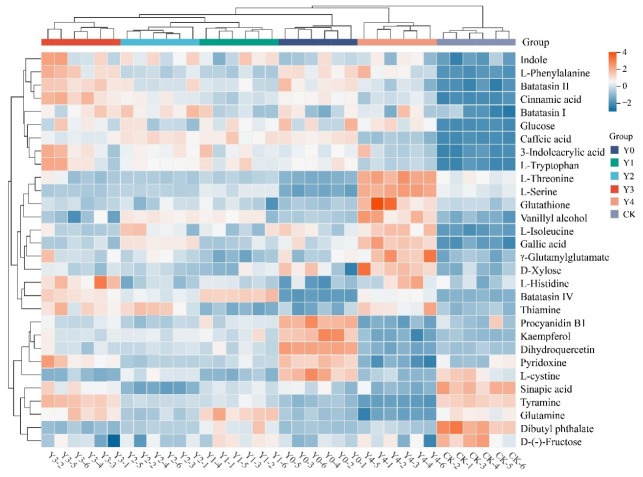
HCA heatmap of *D. opposita* samples across six different years of rotation. In the heatmap, columns represent individual samples, while rows correspond to each metabolite. Note: The color gradient indicates metabolites enrichment levels, ranging from low (blue) to high (red).

Differential metabolites among the six treatment groups (Y0, Y1, Y2, Y3, Y4 and CK) of *D. opposita* were extracted based on the criteria of VIP > 1 and *p* < 0.05. Consequently, 30 differentially accumulated metabolites were screened. Hierarchical clustering analysis (HCA) was performed on these 30 differentially accumulated metabolites across all groups, and the results are presented as a heatmap in Figure 5. Based on their expression profiles, the 30 metabolites were divided into three distinct clusters. Cluster 1 characterizes nine metabolites that were highly expressed in Y3, whereas presented at low levels in CK. Cluster 2 included 11 metabolites that were enriched in Y4. Among these, six were identified as amino acids (such as L-threonine, L-serine, L-histidine, and L-isoleucine), two as phenolic acids (vanillic alcohol and gallic acid). The other three metabolites were saccharides, vitamins and batatasin IV. The third cluster was clustered into two subgroups. One subgroup represented five metabolites, which are named procyanidin B1, kaempferol, dihydroquercetin, pyridoxine, and L-cystine. These compounds were more abundant in Y0 than in the other five treatments. The other subgroup also comprised five metabolites, including sinapic acid, tyramine, glutamine, dibutyl phthalate, and fructose, all of which showed the highest levels in the CK group.

In addition, the six rotation treatments were divided into three clusters. Treatment Y0, Y1, Y2, and Y3 were grouped together. The other two groups Y4 and CK were assigned to separate classes. This clustering pattern was approximately in accordance with the PCA results. It meant that the 30 selected metabolites make more contribution than other components. These findings indicate that these metabolites may serve as pivotal in the quality alterations of *D. opposita* under different crop rotation years.

### 3.4. Correlation Analysis of 30 Differentially Abundant Metabolites and Agronomic Traits

**Figure 6:**
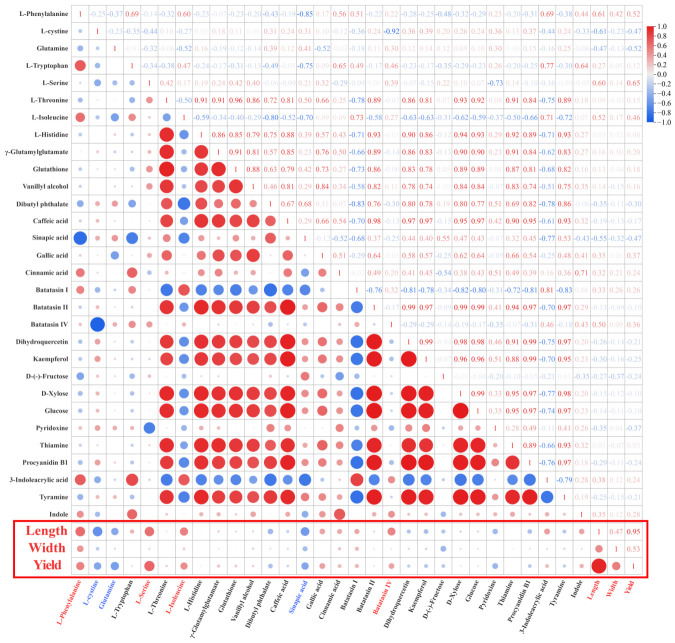
Correlation analysis between 30 differentially abundant metabolites and agronomic traits (tuber length, tuber diameter and tuber yield) of *D. opposita* across six rotation years. Note: The color gradient from blue to red indicates a change in correlation from −1 to 1. Red for the positive correlation, blue for the negative correlation. The larger the graphical elements in the lower triangular region, the stronger the correlation; conversely, smaller elements indicate weaker correlation.

To further elucidate the effects of different metabolites on the agronomic traits of *D. opposita* tubers, a correlation coefficient matrix was constructed between the 30 differentially abundant metabolites and three key agronomic traits (tuber length, tuber diameter and tuber yield) following z-score normalizing of all experimental data (Figure 6). Glucose, L-threonine, batatasin II, caffeic acid, L-threonine, L-histidine, γ-glutamylglutamate, and glutathione showed positive correlations with most other compounds. For example, glucose was extremely positively correlated to L-threonine (0.92), L-histidine (0.93), caffeic acid (0.97), batatasin II (0.99), dihydroquercetin (0.98), kaempferol (0.96), D-xylose (0.99), thiamine (0.95), procyanidin B1 (0.97), and tyramine (0.97). In contrast, batatasin I, 3-indoleacrylic acid and L-isoleucine were negatively correlated with the majority of metabolites. Taking batatasin I as an example, it exhibited negative correlations with L-threonine (−0.78), dibutyl phthalate (−0.83), dihydroquercetin (−0.81), kaempferol (−0.78), D-xylose (−0.82), glucose (−0.80), procyanidin B1 (−0.81), and tyramine (−0.83). The number of positive correlations between metabolites was greater than that of negative correlations.

Regarding metabolite–trait associations, tuber length was positively correlated with L-phenylalanine, L-serine, L-isoleucine, and batatasin IV. The correlation coefficients were as follows: 0.61, 0.6, 0.52, and 0.5. Conversely, tuber length was negatively correlated with L-cystine (−0.61), sinapic acid (−0.55), and glutamine (−0.47). Given that tuber length and tuber yield were strongly and positively correlated with each other (correlation coefficient = 0.95), the set of differential metabolites associated with tuber yield was largely consistent with that related to tuber length. Collectively, these findings indicate that L-phenylalanine, L-serine, L-isoleucine, batatasin IV, L-cystine, sinapic acid, and glutamine serve as critical metabolites, underlying the variations in both tuber length and tuber yield of *D. opposita*.

## 4. Discussion

### 4.1. Effect of Rotation Years on Agronomic Traits, Disease Incidence, and Metabolomics in D. opposita

*D. opposita* is an edible and pharmaceutical food in China. It rich in starch, protein, allantoin, flavonoids, and diosgenin, which are crucial determinants of its nutritional and medicinal quality [31]. During production, *D. opposita* quality is influenced by multiple factors, among which crop rotation years significantly affect plant growth and metabolite profiles [32,33]. However, comprehensive studies on the effects of different rotation years on *D. opposita* growth and quality remain limited. In this study, continuous cropping (Y0) resulted in the highest disease index, shortest tuber length, lowest yield, and severe malformation with excessive branching. These findings align with prior research [4,21,29]. For rotation years of less than four (Y0–Y3), no significant differences in tuber length or yield were observed. The disease index across these years ranged from 40.14 to 77.00, with Y0 and Y1 classified as grade IV severity, and Y2 and Y3 as grade III. These results confirm that rotation years < 4 restrict *D. opposita* growth and reduce yield, exhibiting typical symptoms of continuous cropping obstacles—stunted growth, leaf chlorosis, increased disease incidence, and reduced yield [34]. Thus, *D. opposita* cultivated under rotation durations of less than four years suffers from continuous cropping obstacles. Similar findings in sugar beet (*Beta vulgaris*) indicate that a two-year rotation is insufficient to a substantial increase in sugar yield, recommending a minimum rotation length of three years [32]. Metabolomic analysis revealed distinct metabolite profiles in *D. opposita* tubers across rotation years. A total of 30 differentially accumulated metabolites were screened as potential diagnostic markers. Notably, five metabolites, namely procyanidin B1, kaempferol, dihydroquercetin, pyridoxine, and L-cystine, were more abundant in Y0 than in other treatments. This pattern aligns with findings in *Patchouli*, where continuous cropping induced marked changes in cinnamic acids and flavonoids compared to first-cropping conditions [35]. Collectively, these results demonstrate that *D. opposita* response to continuous cropping stress through dynamic alterations in metabolite composition, with the degree of change variation differing across rotation years.

### 4.2. Chlorophyll Content and Net Photosynthetic Rate (Pn) Were the Primary Factors Driving the Differences in Plant Growth and Tuber Metabolites of D. opposita Across Different Rotation Years

Plant growth, quality, and yield are typically influenced by photosynthesis [36]. As a highly sensitive physiological process, photosynthesis is vulnerable to environmental stressors such as temperature, light intensity, and continuous cropping systems [37]. However, few studies have examined photosynthesis in *D. opposita* under different rotation years. In this study, Y0 (continuous cropping) and Y1 showed consistently lower chlorophyll levels from DAP 90 to DAP 150. By DAP 180, leaves of Y0, Y1, and Y2 had fully senesced with no measurable chlorophyll content, whereas Y4 and CK retained partial chlorophyll. Consistent with our findings, previous studies on *Polygonatum odoratum* [38] and *Pinellia ternate* [39] reported that continuous cropping significantly reduced leaf chlorophyll content and impaired plant growth. Furthermore, *D. opposita* leaves under rotation years of less than four years (Y0–Y3) exhibited markedly diminished net photosynthetic rate (*Pn*), stomatal conductance (*Gs*), and transpiration rate (*Tr*), with *Pn* showing the most pronounced decline compared to Y4 and CK. Similar photosynthetic impairments under different rotation years have been reported in *Rehmannia glutinosa* [40].

Similarly to findings in *Pinellia ternata*, where continuous cropping reduces stomatal density and aperture [39], our study showed that from DAP 120 to harvest, Y0 and Y1 exhibited the lowest *Pn* associated with the lowest *Gs*, restricting CO_2_ supply and utilization. The diminished photosynthetic source capacity restricted plant growth [41]. According to the source–sink theory, source limitation impaired photoassimilate translocation to underground sinks, further exacerbating photosynthetic decline—a key mechanism underlying quality formation in *D. opposita* under different rotation years [42]. Furthermore, soil nitrogen and phosphorus deficiencies inhibit chlorophyll biosynthesis and photosynthetic activity [43]. Thus, the relationship between soil physicochemical properties across rotation years and leaf photosynthetic characteristics in *D. opposita* warrants further investigation.

### 4.3. Flavonoids Emerged as the Key Differential Metabolites in D. opposita in Response to Varying Rotation Years

Secondary metabolites in medicinal and edible homologous plants serve as critical indicators for quality evaluation [44], and specific metabolites play important roles in plant resistance to environmental stress [45]. In this study, 12 metabolites, primarily flavonoids, gradually decreased with increasing rotation years. KEGG enrichment analysis of differential metabolites revealed that flavonoid biosynthesis and flavone/flavonol biosynthesis pathways were enriched across year-pair comparisons. Our findings are consistent with An et al. [9], who reported that flavonoid biosynthesis is among the most active metabolic pathways during tuber development and harvest in *D. opposita*, suggesting that flavonoid metabolism is highly responsive to cultivation practices, including rotation regimes. Similarly, in other tuber crops such as sweet potato (*Ipomoea batatas* L.), flavonoids were identified as the most abundant class of differentially accumulated metabolites (accounting for 23.7–30.3% of total), with KEGG analysis confirming the activation of flavonoid biosynthesis pathways under drought stress [46]. Collectively, these findings indicate that flavonoids serve as the predominant differentially regulated metabolites in response to cropping stress. Using CK as a control, the number of up-regulated flavonoid metabolites in *D. opposita* under rotation years < 4 (Y0–Y3) significantly outnumbered the down-regulated ones. This observation parallels Ma et al. [47], who found that under continuous cropping stress in pea, up-regulated flavonoids outnumbered down-regulated ones, total flavonoid content increased with cropping intensity, and specific isoflavonoids exhibited both antioxidant and antifungal activities. Flavonoids, a class of abundant plant secondary metabolites derived from the phenylpropanoid metabolic pathway, play a crucial role in plant development and defense mechanisms [48] [49]. They act as potent antioxidants that scavenging reactive oxygen species (ROS) generated under biotic and abiotic stress conditions [50]. Thus, the accumulation of flavonoid compounds induced by shorter rotation years plays an important regulatory role in *D. opposita* growth and may help alleviate cropping-induced oxidative stress.

### 4.4. A Four-Year Rotation Is Recommended as the Optimal Length for D. opposita Cultivation

The optimal years of crop rotation serves as the cornerstone of a well-planned and sustainable rotational farming system, directly influencing crop quality [32]. Empirical evidence from diverse crops underscores the critical role of rotation years. For American ginseng, a 3-year maize rotation enhanced replanted root biomass while reducing root disease incidence [51]. Sugar beet rotation length was recommended should be at least three years [32]. Studies on *Panax notoginseng* demonstrate that intervals of ≥3 years optimize plant growth, physiological indices, and saponin accumulation [33,52]. Similarly, *Rehmannia glutinosa*, a member of the *Four huai medicines*, required a 7-year rotation to restore growth indicators, yield, and bioactive compound levels [40]. In this study, 4-year rotation (Y4) exhibited substantial improvements in both aerial growth and tuber quality compared to shorter years (Y0–Y3), as evidenced by increased tuber length and yield. Although Y4 maintained distinct agronomic traits relative to CK (never cultivated yam, interval years ≥ 8), its performance consistently met Grade II quality standards for commercial yams as defined by *National and Geographic Indication Product Standards*. Metabolomic analysis further corroborated these findings: principal component analysis (PCA) and clustering of 30 differential compounds revealed that rotation years < 4 years (Y0–Y3) formed a distinct cluster, significantly diverging from CK and Y4 groups. These results advocate for a 4-year rotation as the optimal strategy for *D. opposita* cultivation. This recommendation aligns with prior studies that *D. opposita* should be planted in soils with ≥3 years of gramineous crop rotation [53] and *Huai Shan Yao* requires a 5-year fallow period between plantings [26].

### 4.5. Limitations and Future Perspectives

Although this study has analyzed the effects of different rotation years on *D. opposita* from three perspectives—agronomic traits, photosynthetic, and tuber metabolomics—several limitations should nonetheless be acknowledged. First, we observed a lower disease index under continuous cropping (Y0) and improved plant performance under longer rotations (especially Y4), suggesting the potential involvement of soil-borne pathogens, particularly root-knot nematodes. This interpretation is supported by studies showing that crop rotation could suppress root-knot nematodes by breaking their life cycles [54]. Importantly, the vertical distribution of *Meloidogyne incognita* is depth dependent. In *D. opposita*, higher abundance was observed at 16–40 cm [55], while in Chinese yam (*Dioscorea batatas*), peak J2 density was reported at 40–50 cm [56]. This poses a challenge for shorter rotations, as deep-residing nematodes may survive and repopulate upon host replanting. Supporting this, research on reniform nematode in cotton found that populations rebounded after a single non-host crop, confirming that one rotation is insufficient and that two or more rotations (i.e., multiple years of non-host cropping) are necessary [57]. These principles align with our finding that a four-year rotation is optimal for *D. opposita*. However, the present study focused on agronomic and metabolomic responses. Future research specifically designed to assess nematode community composition across different rotation years will be essential to elucidate their precise contribution to the observed rotation effects.

Second, soil physicochemical properties and microbial community composition are well-established mediators of crop rotation effects on plant performance [58]. Our investigation did not assess these factors, despite their potential to vary across different rotation years and influence plant growth and metabolic profiles. Third, the underlying mechanisms driving the observed metabolic shifts, such as changes in key enzyme activities involved in secondary metabolic pathways, remain to be elucidated. Future studies integrating soil metagenomics, transcriptomics, targeted enzyme activity assays, and pathogen assessments are warranted to disentangle the direct versus indirect effects of rotation years on *D. opposita* performance.

## 5. Conclusions

This study demonstrates the effects of different rotation years on the growth, photosynthetic characteristics, and tuber metabolites of *D. opposita*. Under continuous cropping (Y0), *D. opposita* plants exhibited the highest disease index, the shortest tuber length, the lowest yield, and severe malformation accompanied by excessive branching. With increasing rotation years, no significant differences in tuber length or yield were observed among Y0, Y1, Y2, and Y3. *D. opposita* leaves subjected to rotation years <4 showed significantly reduced chlorophyll content, *Pn*, *Gs*, and *Tr*. In terms of metabolomics, 30 differentially metabolites were screened. KEGG enrichment analysis revealed all combinations except Y4 vs. CK were enriched in flavonoid and flavone biosynthesis. Flavonoids were identified as the predominant differential metabolites. Collectively, metabolite profiling indicated that the four-year rotation (Y4) represents a critical turning point in the metabolic response of *D. opposita*.

## Figures and Tables

**Table 1:** Identification of 93 metabolites in *D. opposita* across six rotation years by UPLC-MS/MS.

Code	Compounds	Classification	Formula	Molecular Weight (Da)	Observed at *m*/*z* (Da)	RT (min)	Ion Mode	CAS
1	L-Phenylalanine	Amino acids and derivatives	C_9_H_11_NO_2_	165.079	166.09	4.8619	[M + H]^+^	63-91-2
2	L-Cysteine	Amino acids and derivatives	C_3_H_7_NO_2_S	121.0197	121.0296	5.1589	[M − ]^−^	52-90-4
3	L-Cystine	Amino acids and derivatives	C_6_H_12_N_2_O_4_S_2_	240.0238	241.03	5.9805	[M + H]^+^	56-89-3
4	L-Glutamic acid	Amino acids and derivatives	C_5_H_9_NO_4_	147.0532	148.06	0.8474	[M + H]^+^	56-86-0
5	Glutamine	Amino acids and derivatives	C_5_H_10_N_2_O_3_	146.0691	147.0757	0.8622	[M + H]^+^	56-85-9
6	Methionine	Amino acids and derivatives	C_5_H_11_NO_2_S	149.051	150.0576	1.1383	[M + H]^+^	63-68-3
7	Arginine	Amino acids and derivatives	C_6_H_14_N_4_O_2_	174.1117	175.12	0.8082	[M + H]^+^	74-79-3
8	L-Tyrosine	Amino acids and derivatives	C_9_H_11_NO_3_	181.0739	182.0806	1.6255	[M + H]^+^	60-18-4
9	L-Proline	Amino acids and derivatives	C_5_H_9_NO_2_	115.0633	116.07	0.9404	[M + H]^+^	147-85-3
10	L-Tryptophan	Amino acids and derivatives	C_11_H_12_N_2_O_2_	204.0899	205.097	3.1999	[M + H]^+^	73-22-3
11	L-Serine	Amino acids and derivatives	C_3_H_7_NO_3_	105.0426	104.0356	0.8279	[M − H]^−^	56-45-1
12	L-Threonine	Amino acids and derivatives	C_4_H_9_NO_3_	119.0582	120.0655	0.867	[M + H]^+^	72-19-5
13	L-Aspartic acid	Amino acids and derivatives	C_4_H_7_NO_4_	133.0375	132.03	1.1383	[M − H]^−^	56-84-8
14	L-Valine	Amino acids and derivatives	C_5_H_11_NO_2_	117.079	118.09	6.2816	[M + H]^+^	72-18-4
15	L-Isoleucine	Amino acids and derivatives	C_6_H_13_NO_2_	131.0946	132.1016	2.0472	[M + H]^+^	73-32-5
16	L-Histidine	Amino acids and derivatives	C_6_H_9_N_3_O_2_	155.0695	156.08	0.8082	[M + H]^+^	71-00-1
17	γ-Glutamylglutamate	Amino acids and derivatives	C_10_H_16_N_2_O_7_	276.0958	275.1151	0.8474	[M − H]^−^	1116-22-9
18	Glutathione	Amino acids and derivatives	C_10_H_17_N_3_O_6_S	307.0838	306.0746	2.2967	[M − H]^−^	70-18-8
19	Batatasin I	Steroids	C_17_H_16_O_4_	284.1049	285.11	6.7503	[M + H]^+^	51415-00-0
20	Batatasin II	Steroids	C_16_H_18_O_4_	274.12051	275.1275	6.247	[M + H]^+^	39354-56-8
21	Batatasin IV	Steroids	C_15_H_16_O_3_	244.1099	245.117	6.3219	[M + H]^+^	60347-67-3
22	Diosgenin	Steroids	C_27_H_42_O_3_	414.3134	415.3203	7.4204	[M + H]^+^	512-04-9
23	Ferulic acid	Phenolic acids	C_10_H_10_O_4_	194.0579	193.05	4.5952	[M − H]^−^	537-98-4
24	Benzoic acid	Phenolic acids	C_7_H_6_O_2_	122.0368	123.0435	1.3554	[M + H]^+^	65-85-0
25	Syringic acid	Phenolic acids	C_9_H_10_O_5_	198.0528	197.0456	4.4845	[M − H]^−^	530-57-4
26	Vanillyl alcohol	Phenolic acids	C_8_H_10_O_3_	154.063	137.0589	4.7238	[M + H − H_2_O]^+^	498-00-0
27	Sinapic acid	Phenolic acids	C_11_H_12_O_5_	224.0685	223.061	5.0596	[M − H]^−^	530-59-6
28	Caffeic acid	Phenolic acids	C_9_H_8_O_4_	180.0423	179.03	4.6252	[M − H]^−^	331-39-5
29	Pyrocatechol	Phenolic acids	C_6_H_6_O_2_	110.0368	109.03	4.5457	[M − H]^−^	120-80-9
30	Dibutyl phthalate	Phenolic acids	C_16_H_22_O_4_	278.1518	279.1595	7.395	[M + H]^+^	84-74-2
31	Chlorogenic Acid	Phenolic acids	C_16_H_18_O_9_	354.0951	353.0868	3.8605	[M − H]^−^	327-97-9
32	Gallic acid	Phenolic acids	C_7_H_6_O_5_	170.0215	169.01	1.6847	[M − H]^−^	149-91-7
33	Rosmarinic acid	Phenolic acids	C_18_H_16_O_8_	360.0845	359.0768	5.2184	[M − H]^−^	20283-92-5
34	Cinnamic acid	Phenolic acids	C_9_H_8_O_2_	148.0524	149.06	4.8807	[M + H]^+^	140-10-3
35	Vanillic acid	Phenolic acids	C_8_H_8_O_4_	168.0423	167.0349	4.4017	[M − H]^−^	121-34-6
36	Inosine	Nucleotides and derivatives	C_10_H_12_N_4_O_5_	268.0808	269.051	3.7004	[M + H]^+^	58-63-9
37	Guanosine	Nucleotides and derivatives	C_10_H_13_N_5_O_5_	283.0917	284.0989	2.4048	[M + H]^+^	118-00-3
38	Uracil	Nucleotides and derivatives	C_4_H_4_N_2_O_2_	112.0273	111.02	2.2256	[M − H]^−^	66-22-8
39	Adenosine	Nucleotides and derivatives	C_10_H_13_N_5_O_4_	267.0968	266.09	2.5848	[M − H]^−^	58-61-7
40	Adenine	Nucleotides and derivatives	C_5_H_5_N_5_	135.0545	134.0476	2.658	[M − H]^−^	73-24-5
41	Epicatechin	Flavonoids	C_15_H_14_O_6_	290.079	291.09	4.3818	[M + H]^+^	490-46-0
42	Cianidanol	Flavonoids	C_15_H_14_O_6_	290.079	289.07	4.0527	[M − H]^−^	154-23-4
43	Quercetin	Flavonoids	C_15_H_10_O_7_	302.0427	303.0499	4.8226	[M − H]^−^	117-39-5
44	Rutin	Flavonoids	C_27_H_30_O_16_	610.1534	609.1456	4.7608	[M − H]^−^	153-18-4
45	Dihydroquercetin	Flavonoids	C_15_H_12_O_7_	304.0583	303.0502	4.3818	[M − H]^−^	480-18-2
46	Apigenin	Flavonoids	C_15_H_10_O_5_	270.0528	271.0601	5.8597	[M + H]^+^	520-36-5
47	Kaempferol	Flavonoids	C_15_H_10_O_6_	286.0477	285.0398	4.1418	[M − H]^−^	520-18-3
48	Myricetin	Flavonoids	C_15_H_10_O_8_	318.0376	319.0449	4.5853	[M + H]^+^	529-44-2
49	Procyanidin B1	Flavonoids	C_30_H_26_O_12_	578.1424	577.14	4.1017	[M − H]^−^	20315-25-7
50	3,4-Dihydrocoumarin	Coumarins	C_9_H_8_O_2_	148.0524	147.05	2.3368	[M − H]^−^	119-84-6
51	Resveratrol	Others	C_14_H_12_O_3_	228.0786	227.0713	5.4579	[M − H]^−^	501-36-0
52	5-Hydroxymethyl-2-furancarboxaldehyde	Others	C_6_H_6_O_3_	126.0317	127.0382	0.9204	[M + H]^+^	67-47-0
53	Arabinose	Saccharides	C_5_H_10_O_5_	150.0528	149.0459	6.4139	[M − H]^−^	10323-20-3
54	D-Mannose	Saccharides	C_6_H_12_O_6_	180.0634	217.0028	3.138	[M + K − 2H]^−^	530-26-7|46032-76-2
55	D-(-)-Fructose	Saccharides	C_6_H_12_O_6_	180.0634	179.06	0.8867	[M − H]^−^	57-48-7
56	D-Xylose	Saccharides	C_5_H_10_O_5_	150.0528	166.0771	2.7583	[M + NH_4_ − 2H]^−^	58-86-6
57	Glucuronic Acid	Saccharides	C_6_H_10_O_7_	194.0427	193.0352	0.9064	[M − H]^−^	528-16-5
58	Cellobiose	Saccharides	C_12_H_22_O_11_	342.1162	341.12	5.5188	[M − H]^−^	528-50-7
59	Sucrose	Saccharides	C_12_H_22_O_11_	342.1162	341.11	1.2173	[M − H]^−^	57-50-1
60	Maltose	Saccharides	C_12_H_22_O_11_	342.1162	341.11	0.9603	[M − H]^−^	69-79-4
61	Maltohexaose	Saccharides	C_36_H_62_O_31_	990.3275	989.3188	2.4773	[M − H]^−^	34620-77-4
62	Maltotriose	Saccharides	C_18_H_32_O_16_	504.169	527.1576	1.2173	[M + Na]^+^	1109-28-0
63	Glucose	Saccharides	C_6_H_12_O_6_	180.0634	359.1236	2.6982	[2M − H]^−^	50-99-7
64	Gluconic acid	Saccharides	C_6_H_12_O_7_	196.0583	195.0515	0.867	[M − H]^−^	526-95-4
65	Rhamnose	Saccharides	C_6_H_12_O_5_	164.0685	163.0612	0.6897	[M − H]^−^	3615-41-6
66	Cellotriose	Saccharides	C_18_H_32_O_16_	504.169	485.1497	2.5573	[M − H_2_O − H]^−^	9061-30-7
67	Pyridoxine	Vitamin	C_8_H_11_NO_3_	169.0739	170.0805	1.8345	[M + H]^+^	65-23-6
68	Thiamine	Vitamin	C_12_H_17_N_4_OS+	265.1123	265.1114	1.4741	[M]^+^	70-16-6
69	Nicotinic acid	Vitamin	C_6_H_5_NO_2_	123.032	124.0387	0.9603	[M + H]^+^	59-67-6
70	Niacinamide	Vitamin	C_6_H_6_N_2_O	122.048	123.0548	2.3652	[M + H]^+^	98-92-0
71	Folic acid	Vitamin	C_19_H_19_N_7_O_6_	441.1397	459.1785	6.1803	[M + NH_4_]^+^	59-30-3
72	3-Indoleacrylic acid	Alkaloids	C_11_H_9_NO_2_	187.0633	188.0707	3.1999	[M + H]^+^	29953-71-7
73	Tyramine	Alkaloids	C_8_H_11_NO	137.0841	138.0906	1.5532	[M + H]^+^	51-67-2
74	Choline	Alkaloids	C_5_H_14_NO+	104.1075	104.1064	0.8426	[M]^+^	62-49-7
75	Moupinamide	Alkaloids	C_18_H_19_NO_4_	313.1314	314.139	5.4989	[M + H]^+^	66648-43-9
76	p-Coumaroyltyramine	Alkaloids	C_17_H_17_NO_3_	283.1208	284.1282	5.4385	[M + H]^+^	36417-86-4
77	Dopamine	Alkaloids	C_8_H_11_NO_2_	153.079	154.0855	1.3522	[M + H]^+^	51-61-6
78	Trigonelline	Alkaloids	C_7_H_7_NO_2_	137.0477	138.0548	0.9404	[M + H]^+^	535-83-1
79	Ecgonine methyl ester	Alkaloids	C_10_H_17_NO_3_	199.1208	238.0915	1.5728	[M + K]^+^	7143-09-1
80	Indole	Alkaloids	C_8_H_7_N	117.0578	118.0646	3.1999	[M + H]^+^	120-72-9
81	Arbutin	Terpenoids	C_12_H_16_O_7_	272.0896	271.0821	3.8004	[M − H]^−^	84380-01-8
82	Abscisic acid	Organic acids	C_15_H_20_O_4_	264.1362	263.1284	5.6577	[M − H]^−^	21293-29-8
83	2-Hydroxyglutarate	Organic acids	C_5_H_8_O_5_	148.0372	147.0298	1.1611	[M − H]^−^	13095-48-2
84	4-Hydroxybutyric acid	Organic acids	C_4_H_8_O_3_	104.0473	149.0232	7.395	[M − H + 2Na]^+^	591-81-1
85	Malic acid	Organic acids	C_4_H_6_O_5_	134.0215	133.0149	1.5867	[M − H]^−^	636-61-3
86	Pantothenic acid	Organic acids	C_9_H_17_NO_5_	219.1107	218.11	3.0581	[M − H]^−^	79-83-4
87	γ-Aminobutyric Acid	Organic acids	C_4_H_9_NO_2_	103.0633	104.07	0.8426	[M + H]^+^	56-12-2
88	Succinic acid	Organic acids	C_4_H_6_O_4_	118.0266	117.02	2.1964	[M − H]^−^	110-15-6
89	Citric acid	Organic acids	C_6_H_8_O_7_	192.027	191.0205	1.1611	[M − H]^−^	77-92-9
90	Azelaic acid	Organic acids	C_9_H_16_O_4_	188.1049	187.0976	5.2982	[M − H]^−^	123-99-9
91	Robustic acid	Organic acids	C_22_H_20_O_6_	380.126	379.1224	3.5155	[M − H]^−^	5307-59-5
92	Stearic acid	Lipids	C_18_H_36_O_2_	284.2715	283.2644	7.9719	[M − H]^−^	57-11-4
93	Oleic acid	Lipids	C_18_H_34_O_2_	282.2559	265.2523	7.1972	[M + H − H_2_O]^+^	112-80-1

## Data Availability

The data presented in this study are available within the article and its Supplementary Materials. The data that support the findings of this study are available from the corresponding author upon reasonable request.

## Supplementary Materials

The following supporting information can be downloaded at: https://www.mdpi.com/article/10.3390/metabo16070492/s1, Table S1: List of the differential metabolites of *D. opposita* among the comparison groups; Figure S1: Experimental design and treatment configurations; Figure S2: Proportions of the 93 metabolites identified from *D. opposita* samples (A) and PCA score plot (B).
