# Supplementary material for: Six Rotation Years Drive Dynamic Shifts in Agronomic Traits, Photosynthesis, and Tuber Metabolomics of Dioscorea opposita Thunb
Source: Metabolites. 2026 Jul 13;16(7):492. doi: 10.3390/metabo16070492 (PMC13413904; doi:10.3390/metabo16070492)
Supplement: Supplementary file 1 [file metabolites-16-00492-s001.zip › metabolites-4309770-supplementary.pdf]

Supplementary Materials

Table S1. List of the differential metabolites of *D. opposita* among the comparison groups

Group	Compounds	Class	VIP	P-value	FDR	Fold_Change	Log2FC	Type
Y0 VS CK	L-Tyrosine	Amino acids	1.147	0.001	0.002	2.241	1.164	up
	Caffeic acid	Phenolic acids	1.224	0.000	0.000	4.198	2.070	up
	Rosmarinic acid	Phenolic acids	1.222	0.000	0.000	2.421	1.275	up
	Cinnamic acid	Phenolic acids	1.224	0.000	0.000	2.082	1.058	up
	Epicatechin	Flavonoids	1.223	0.000	0.000	2.811	1.491	up
	Cianidanol	Flavonoids	1.137	0.003	0.005	2.004	1.003	up
	Quercetin	Flavonoids	1.227	0.000	0.000	6.626	2.728	up
	Rutin	Flavonoids	1.184	0.000	0.000	2.404	1.265	up
	Dihydroquercetin	Flavonoids	1.223	0.000	0.000	2.042	1.030	up
	Myricetin	Flavonoids	1.227	0.000	0.000	8.390	3.069	up
	Arabinose	Saccharides	1.211	0.000	0.000	3.164	1.662	up
	Glucose	Saccharides	1.200	0.001	0.003	10.121	3.339	up
	Folic acid	Vitamin	1.148	0.000	0.000	0.345	-1.536	down
	Moupinamide	Phenolamine	1.196	0.000	0.000	0.444	-1.173	down
	L-Cysteine	Amino acids	1.225	0.000	0.000	0.253	-1.981	down
	L-Glutamic acid	Amino acids	1.225	0.000	0.000	0.468	-1.096	down
	L-Serine	Amino acids	1.201	0.000	0.000	0.422	-1.246	down
	Dibutyl phthalate	Phenolic acids	1.198	0.000	0.000	0.389	-1.361	down
	Resveratrol	others	1.220	0.000	0.000	0.385	-1.375	down
Y1 VS CK	Caffeic acid	Phenolic acids	1.348	0.000	0.000	4.157	2.055	up
	Quercetin	Flavonoids	1.353	0.000	0.000	2.363	1.241	up
	Myricetin	Flavonoids	1.334	0.000	0.000	2.115	1.080	up
	Arabinose	Saccharides	1.236	0.002	0.004	2.035	1.025	up
	Glucose	Saccharides	1.343	0.000	0.000	9.615	3.265	up
	Ecgonine methyl ester	Tropan alkaloids	1.066	0.014	0.024	0.449	-1.155	down
Y2 VS CK	Benzoic acid	Phenolic acids	1.232	0.005	0.011	3.296	1.721	up
	Caffeic acid	Phenolic acids	1.325	0.000	0.000	4.262	2.092	up
	Dibutyl phthalate	Phenolic acids	1.285	0.000	0.001	0.451	-1.148	down
	Cinnamic acid	Phenolic acids	1.287	0.000	0.001	2.245	1.167	up
	Quercetin	Flavonoids	1.322	0.000	0.000	4.311	2.108	up
	Rutin	Flavonoids	1.063	0.024	0.040	2.707	1.437	up
	Myricetin	Flavonoids	1.324	0.000	0.000	4.744	2.246	up
	Arabinose	Saccharides	1.313	0.000	0.000	3.941	1.979	up

	Glucose	Saccharides	1.283	0.002	0.005	8.860	3.147	up
	Thiamine	Vitamin	1.111	0.017	0.029	2.380	1.251	up
	Dopamine	Phenolamine	1.257	0.003	0.006	3.217	1.686	up
Y3 VS CK	Caffeic acid	Phenolic acids	1.355	0.000	0.000	3.559	1.831	up
	Dibutyl phthalate	Phenolic acids	1.325	0.000	0.000	0.362	-1.466	down
	Cinnamic acid	Phenolic acids	1.344	0.000	0.000	2.415	1.272	up
	Quercetin	Flavonoids	1.356	0.000	0.000	4.732	2.242	up
	Rutin	Flavonoids	1.332	0.000	0.000	2.703	1.435	up
	Myricetin	Flavonoids	1.359	0.000	0.000	5.612	2.489	up
	Arabinose	Saccharides	1.164	0.014	0.026	2.931	1.551	up
	Maltotriose	Saccharides	1.355	0.000	0.000	3.721	1.896	up
	Glucose	Saccharides	1.337	0.000	0.001	9.301	3.217	up
	p-Coumaroyltyramine	Phenolamine	1.349	0.000	0.000	2.853	1.512	up
Y4 VS CK	Glutathione	Amino acids	1.185	0.008	0.016	2.244	1.166	up
	Caffeic acid	Phenolic acids	1.342	0.000	0.000	2.449	1.292	up
	Dibutyl phthalate	Phenolic acids	1.301	0.000	0.000	0.461	-1.118	down
	Glucose	Saccharides	1.353	0.000	0.000	10.051	3.329	up
	Tyramine	Alkaloids	1.361	0.000	0.000	0.383	-1.384	down
	p-Coumaroyltyramine	Phenolamine	1.357	0.000	0.000	0.212	-2.238	down
Y0 VS Y1	L-Cysteine	Amino acids	1.411	0.000	0.000	0.338	-1.563	down
	L-Serine	Amino acids	1.393	0.000	0.000	0.433	-1.206	down
	Batatasin IV	Phenolic acids	1.415	0.000	0.000	0.394	-1.343	down
	Quercetin	Flavonoids	1.413	0.000	0.000	2.804	1.487	up
	Myricetin	Flavonoids	1.410	0.000	0.000	3.967	1.988	up
	Resveratrol	Others	1.415	0.000	0.000	0.288	-1.794	down
	Folic acid	Vitamin	1.360	0.000	0.000	0.257	-1.959	down
	p-Coumaroyltyramine	Phenolamine	1.408	0.000	0.000	0.425	-1.235	down
	Diosgenin	Steroid	1.040	0.002	0.006	0.307	-1.703	down
Y1 VS Y2	Benzoic acid	Phenolic acids	1.335	0.011	0.026	0.423	-1.241	down
	Myricetin	Flavonoids	1.508	0.000	0.000	0.446	-1.166	down
	Thiamine	Vitamin	1.319	0.011	0.026	0.359	-1.477	down
	Dopamine	Phenolamine	1.389	0.005	0.017	0.418	-1.258	down
	Maltotriose	Saccharides	1.746	0.000	0.000	0.299	-1.740	down

Y2 VS Y3	p-Coumaroyltyramine	Phenolamine	1.696	0.000	0.000	0.481	-1.055	down
Y3 VS Y4	L-Tyrosine	Amino acids	1.102	0.015	0.026	2.218	1.149	up
	L-Serine	Amino acids	1.360	0.000	0.000	0.432	-1.212	down
	Glutathione Reducedform	Amino acids	1.192	0.008	0.016	0.449	-1.154	down
	Ferulic acid	Phenolic acids	1.298	0.000	0.001	2.361	1.239	up
	Rosmarinic acid	Phenolic acids	1.315	0.000	0.000	2.096	1.068	up
	Quercetin	Flavonoids	1.359	0.000	0.000	4.495	2.168	up
	Rutin	Flavonoids	1.322	0.000	0.000	2.611	1.384	up
	Myricetin	Flavonoids	1.357	0.000	0.000	4.343	2.119	up
	Resveratrol	Others	1.356	0.000	0.000	0.426	-1.231	down
	Cellobiose	Saccharides	1.282	0.000	0.001	0.392	-1.353	down
	Maltotriose	Saccharides	1.360	0.000	0.000	4.940	2.304	up
	Gluconic acid	Saccharides	1.360	0.000	0.000	2.049	1.035	up
	Pyridoxine	Vitamin	1.260	0.000	0.000	2.124	1.087	up
	Folic acid	Vitamin	1.249	0.000	0.000	2.103	1.073	up
	Tyramine	Alkaloids	1.361	0.000	0.000	2.702	1.434	up
	p-Coumaroyltyramine	Phenolamine	1.361	0.000	0.000	13.459	3.751	up

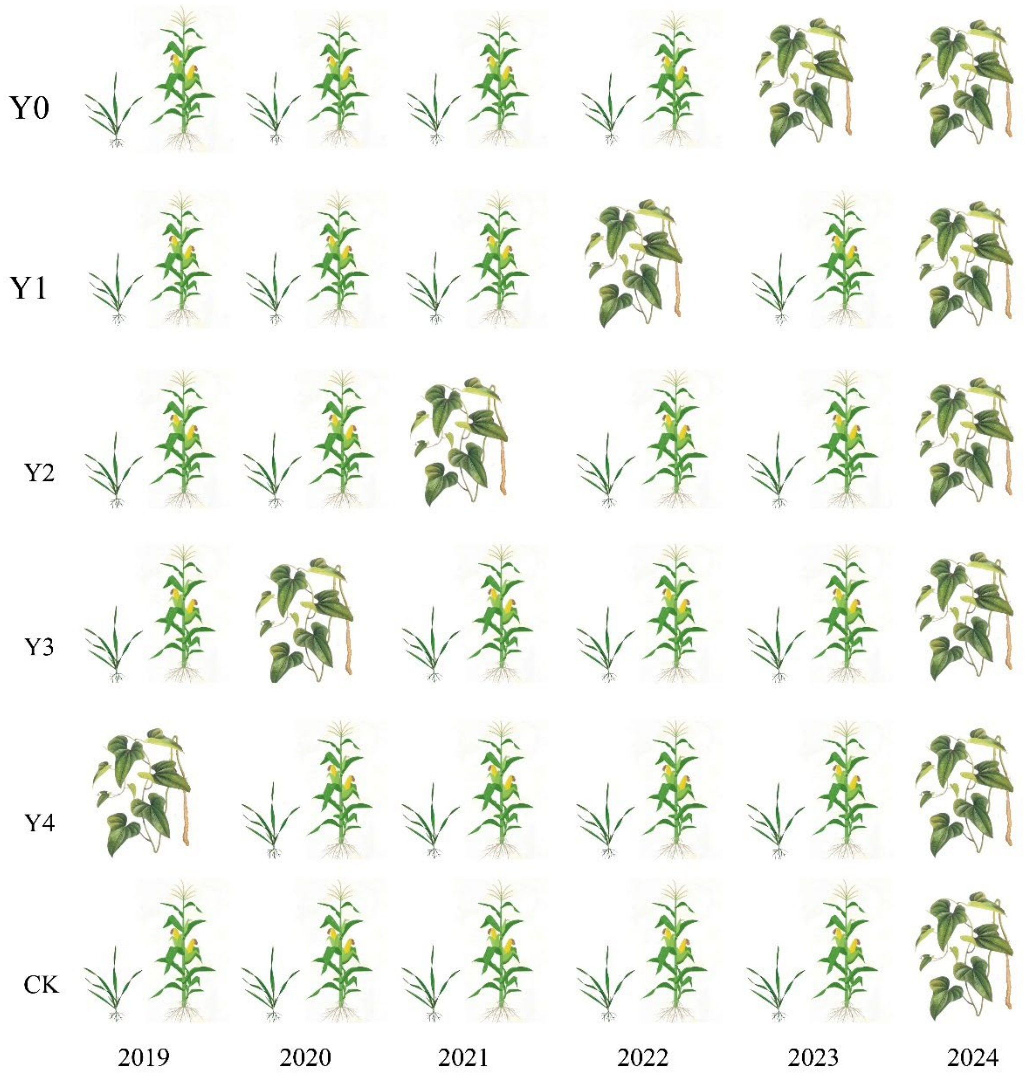

Figure S1. Experimental design and treatment configurations

Note: Y0 (0-year rotation, continuous cropping), Y1 (1-year rotation), Y2 (2-year rotation), Y3 (3-year rotation), Y4 (4-year rotation), and CK (uncultivated *D. opposita* land with rotation years ≥8)

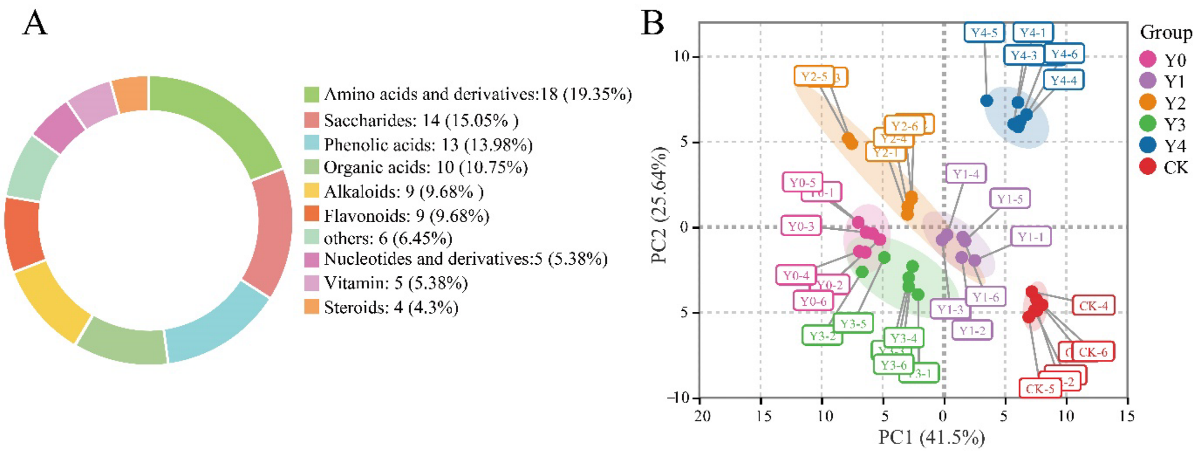

Figure S2. Proportions of the 93 metabolites identified from *D. opposita* samples (A) and PCA score plot (B).

Note: The "others" category includes all groups with fewer than four compounds.
